# Benchmarking Deep Learning Predictions of Mutation-Induced Fold Switching

**DOI:** 10.64898/2026.08.02.742283

**Published:** 2026-08-02

**Authors:** Nathaniel Felbinger, Kathleen Joyce Carillo, Yihong Chen, John Orban, Brian G. Pierce

## Abstract

Many proteins are known to adopt multiple distinct folded states which are often associated with key functional behavior. A predictive understanding of the properties of such fold-switching or metamorphic proteins can provide insights into protein dynamics and energetics, and enable the design of complex protein functions and molecular machines. Recently developed deep learning modeling tools, including AlphaFold, have led to dramatic increases in accuracy for prediction of protein structures from sequence, but their performance for prediction of point mutant effects or fold switching is unclear. Here we present a systematic NMR-characterized dataset of mutants of the GA/GB model fold-switching system and use it to evaluate whether current structure prediction and design methods can predict mutation-induced changes in fold state. We measured fold-state populations for variants at three key positions that differentially stabilize the 3α, 4β+α, mixed, or unfolded states, generating a quantitative experimental benchmark for mutation-level fold switching. Using this benchmark to assess and compare a panel of deep learning and physics-based modeling and design algorithms, we found that this benchmark revealed variable and position-dependent performance across methods, with certain AlphaFold2-based algorithms were able to predict mutant effects at individual sites, indicating some understanding of physical effects of residue substitutions. Additional comparisons of predictions with experimentally measured stability changes further highlighted position-dependent success and general challenges for predictive algorithms. Together, this study provides a new benchmark for mutation-induced fold switching and reveals the current capabilities and limitations of deep learning models for predicting mutation-dependent protein conformational states.

## Full Text Availability

The license terms selected by the author(s) for this preprint version do not permit archiving in PMC. The full text is available from the preprint server.

